# Supplementary material for: Soil Zinc Is Associated with Serum Zinc But Not with Linear Growth of Children in Ethiopia
Source: Nutrients. 2019 Jan 22;11(2):221. doi: 10.3390/nu11020221 (PMC6413067; doi:10.3390/nu11020221)
Supplement: Supplementary file 1 [file nutrients-11-00221-s001.pdf]

Table S1. Multi-level mixed linear regression model predicting serum zinc: subgroup analyses by age [1].

	**Model 1 (Children ≥2 4 months)**			**Model 2 (Children ≤ 23 months)**		
**Fixed effects**	**Estimate**	**SE**	***p***	**Estimate**	**SE**	***p***
Soil zinc (mg/kg)	0.8	0.4	0.043	1.8	1.1	0.099
Diarrhea in past two weeks	−0.8	2.1	0.692	−6.1	3.8	0.107
CRP (mg/L) and AGP (g/L) (ref=normal)						
Elevated CRP only (mg /L)	−1.6	7.4	0.827	−11.5	15.1	0.446
Elevated AGP only (g/L)	−4.8	1.5	0.002	−0.1	3.5	0.972
Elevated AGP (g/L) and CRP (mg/L)	−7.0	2.3	0.002	−6.8	5.0	0.175
Sex of child (female)	−2.1	1.4	0.127	0.3	3.2	0.918
Wealth status (Ref = wealthier)						
Wealth status (poorer)	1.6	1.9	0.402	4.3	4.1	0.293
Wealth status (medium)	2.2	1.8	0.223	7.7	4.0	0.052
Time since most recent meal (hr)	−4.9	3.6	0.173	−18.2	8.8	0.040
FIES [2]	−0.2	0.3	0.363	0.6	0.6	0.343
Consumption of meat or meat products in the last 24h	−2.0	2.1	0.345	−17.2	6.9	0.014
**Random effects**						
Intercept (cluster)	32.4	13.4	0.008	0.0		

[1] Models adjusted for regions as fixed effects. The restricted maximum likelihood (REML) method was used to estimate the parameters. [2] FIES = The Food Insecurity Experience Scale.

Table S2. Multi-level mixed linear regression models predicting height-for-age Z-score: subgroup analyses by age [1].

	Model 1 (Children ≥ 24 months)			Model 2 (Children ≤ 23 months)		
**Fixed effects**	**Estimate**	**SE**	***p***	**Estimate**	**SE**	***p***
Soil zinc (mg/kg)	0.017	0.034	0.612	0.056	0.121	0.645
Serum zinc (μg/dL)	0.000	0.003	0.941	−0.008	0.008	0.295
Diarrhea in past two weeks	−0.423	0.191	0.027	−0.158	0.375	0.675
CRP (mg/L) and AGP (g/L) (ref=normal)						
Elevated CRP only (mg /L)	−0.247	0.670	0.713	−0.503	1.481	0.735
Elevated AGP only (g/L)	−0.044	0.141	0.755	0.041	0.345	0.907
Elevated AGP (g/L) and CRP (mg/L)	−0.050	0.207	0.808	0.130	0.453	0.774
Sex of child (female)	0.220	0.125	0.078	0.428	0.298	0.153
Wealth status (Ref = wealthier)						
Wealth status (poorer)	−0.072	0.177	0.683	−0.252	0.409	0.538
Wealth status (medium)	−0.055	0.161	0.734	−0.384	0.385	0.319
FIES [2]	−0.023	0.023	0.329	−0.056	0.059	0.345
**Random effects**						
Intercept (cluster)	0.245	0.100	0.007	1.253	0.570	0.014

[1] Models adjusted for regions as fixed effects. The restricted maximum likelihood (REML) method was used to estimate the parameters. [2] FIES = The Food Insecurity Experience Scale.

Table S3. Multi-level mixed linear regression model predicting serum zinc: subgroup analyses by residence [1].

	Model 1 (Urban)			Model 2 (Rural)		
**Fixed effects**	**Estimate**	**SE**	***p***	**Estimate**	**SE**	***p***
Soil zinc (mg/kg)	2.7	1.0	0.008	0.9	0.3	0.006
Diarrhea in past two weeks	−0.3	5.8	0.957	−1.1	1.9	0.580
CRP (mg/L) and AGP (g/L) (ref=normal)						
Elevated CRP only (mg /L)	−14.6	20.2	0.472	−2.5	7.0	0.719
Elevated AGP only (g/L)	−10.9	3.7	0.004	−2.7	1.5	0.079
Elevated AGP (g/L) and CRP (mg/L)	−9.7	5.6	0.087	−5.3	2.2	0.015
Sex of child (female)	−3.7	3.5	0.293	−2.0	1.4	0.139
Wealth status (Ref = wealthier)						
Wealth status (poorer)	6.1	7.0	0.385	2.7	2.0	0.190
Wealth status (medium)	−2.7	6.2	0.658	3.5	1.9	0.062
Age in months (Ref=6–11 months)						
Age category 2 (12–23 months)	−10.1	9.5	0.291	−0.2	3.7	0.947
Age category 3 (24–59 months)	−10.7	8.5	0.208	0.4	3.3	0.910
Time since most recent meal (hr)	−18.5	8.6	0.034	−2.6	3.5	0.464
FIES [2]	0.2	0.7	0.786	0.0	0.3	0.870
Consumption of meat or meat products in the last 24h	−0.6	4.5	0.894	−3.3	2.2	0.143
**Random effects**						
Intercept (cluster)	0.0			43.6	13.8	0.001

[1] As region and urban/rural designation are closely related in Ethiopia, region was omitted from model. The restricted maximum likelihood (REML) method was used to estimate the parameters. [2] FIES = The Food Insecurity Experience Scale.
